# CRISPR sabotage

**DOI:** 10.1186/s13059-015-0820-0

**Published:** 2015-11-09

**Authors:** John van der Oost, Stan J. J. Brouns

**Affiliations:** Laboratory of Microbiology, Wageningen University, Dreijenplein, 6703 HB Wageningen, The Netherlands

## Abstract

The biological arms race generally involves the rapid co-evolution of anti-virus systems in host organisms and of anti-anti-virus systems in their viral parasites. The CRISPR-Cas system is an example of a prokaryotic immune system in which such co-evolution occurs, as was recently demonstrated by the characterization of a set of viral anti-CRISPR proteins.

## Introduction

Viruses are mobile genetic elements that rely on infecting cellular organisms (eukaryotes or prokaryotes) for replication and proliferation. These viral invasions often reduce the fitness of their host, sometimes leading to host death. This potential threat generates a selective pressure on host organisms to evolve systems that neutralize viral infections. When a protective barrier is successfully established, the pressure to survive is placed back on the parasite. After the rapidly evolving virus has found a way to counteract the defense barrier, the host has to start all over again. The continuous development and adjustment of appropriate infection and resistance strategies results in a rapid co-evolution of viral offence systems and host defense systems. Such a biological arms race implies that never-ending evolution is required for both predator and prey to maintain a constant fitness level; this situation has been described in evolutionary biology as the Red Queen hypothesis [1, 2].

## Interference systems and suppression of RNA interference

To counteract invasions by pathogenic viruses, many vertebrate animals possess adaptive immune systems consisting of specific antibody proteins, whereas many plants and invertebrate animals use RNA-guided RNA interference (RNAi) systems that efficiently recognize and neutralize invading RNA. Likewise, a range of different defense systems to counteract viral attack have been discovered in prokaryotes, both bacteria and archaea. The best-characterized prokaryotic innate immune system concerns restriction/modification (R/M) enzymes. During the past decade, new classes of bacterial defense systems have been discovered that are based on RNA or DNA interference. A ground-breaking discovery has been the elucidation of CRISPR-Cas (clusters of regularly interspaced palindromic repeats and associated proteins), an adaptive immunity system in bacteria and archaea [3]. The CRISPR-Cas system acquires short DNA sequences from invading genetic elements, and stores them in CRISPR arrays in the host genome. Upon an infection by a previously encountered intruder, the CRISPR memory is expressed as small CRISPR RNAs (crRNAs) that guide surveillance complexes to complementary invading nucleic acids, eventually resulting in neutralization of the invasion. CRISPR-Cas systems are classified either as Class-1 systems, which have multi-subunit crRNA–effector complexes (e.g., Cascade and CMR), or as Class-2 systems, which have single protein crRNA–effector complexes (e.g., Cas9 and Cpf1) [4].

**Fig. 1:**
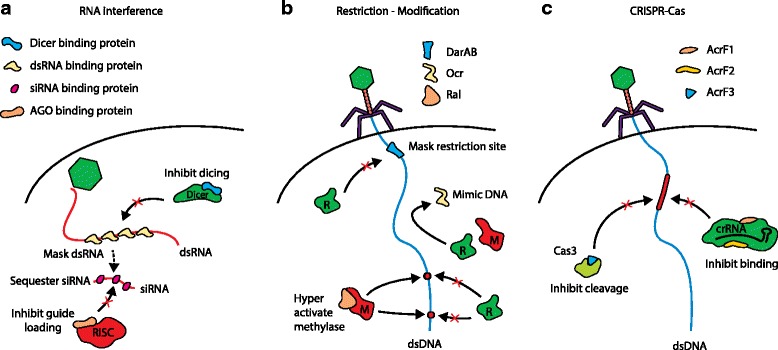
Virus-encoded inhibitors of antiviral defense systems. **a** RNA viruses that infect various plants, insects and mammals encode proteins that inhibit essential proteins in the RNA interference pathway, such as Dicer and Argonaute (*AGO*), a subunit of the RNA-induced silencing complex (*RISC*). Furthermore, these viruses may produce proteins that will mask double-stranded RNA (*dsRNA*) so that Dicer activity does not occur or that will sequester any small interfering RNA (*siRNA*) molecules produced. **b** Bacteriophage P1 co-injects DarA and DarB to mask restriction sites in the phage genome. Bacteriophage T7 encodes Ocr, which mimics the DNA phosphate backbone and sequesters both the *Eco*KI restriction enzyme (*R*) and its corresponding methylase (*M*). Bacteriophage Lambda encodes Ral, which hyperactivates the DNA methylase to protect phage DNA before it can be restricted. **c** Anti-CRISPR proteins encoded by *Pseudomonas* prophages (AcrF1–AcrF3) bind CRISPR-RNA–effector complexes and the nuclease Cas3 to prevent them from binding and cleaving target dsDNAs

Many eukaryotic viruses — for example, plant and insect viruses — carry suppressors of RNAi on their viral genomes to sabotage the RNAi immune system (Fig. 1a). The mechanisms that these suppressors employ are very diverse, ranging from the inhibition of small interfering RNA (siRNA) production, to the formation of unproductive siRNA, sequestering of host siRNA, interference with host gene regulation, and direct inhibition or inactivation of RNAi protein components [5–7].

## Suppression of restriction/modification systems and anti-CRISPR proteins

Several bacteriophages encode small proteins that inhibit or modulate the activity of restriction and DNA modification systems [8] (Fig. 1b). Proteins such as DarA and DarB from bacteriophage P1 are co-injected with phage DNA into *Escherichia coli* cells to protect sensitive restriction sites immediately upon entry. Bacteriophage T7 encodes the Ocr protein, the structure of which mimics double-stranded DNA, and sequesters both the restriction endonuclease *Eco*KI and its corresponding methylase. Bacteriophage Lambda employs a strategy of producing a protein called Ral that activates the host’s DNA methylase to provide rapid protection from restriction.

Recently, dedicated viral proteins have been identified that suppress CRISPR immunity (reviewed by Wiedenheft [6]). Analyses of *Pseudomonas aeruginosa*-specific phages have resulted in the identification of a range of anti-CRISPR (Acr) protein variants [9–11]. Acr proteins were initially discovered by analysis of *Pseudomonas* strains that contain prophages in their chromosome. Although most of these lysogenic strains have a functional Type I-F CRISPR-Cas system (and thus are phage resistant), some of these systems appeared to be inactive, even in the presence of phage-targeting spacers. Molecular analyses of the inactive strains revealed a number of small phage-encoded proteins that were responsible for the observed phage-sensitive phenotype [9]. In a recent follow-up study, it was demonstrated that three selected Acr proteins inhibit the Type I-F CRISPR-Cas system through different mechanisms (Fig. 1c): two suppressors bind to different subunits of the Cascade-like complex to block target DNA binding, whereas the third Acr binds the Cas3 protein to prevent nuclease-helicase activity that is required for target DNA degradation [10]. The tested Acr proteins are highly specific for the *Pseudomonas* I-F system; no suppression was observed in the *E. coli* I-F system or in the *Pseudomonas* I-E system. A separate study [11] revealed that some of the *Pseudomonas* prophages that possess I-F suppressor genes also encode small suppressor proteins that specifically target the *Pseudomonas* I-E system, but not the *E. coli* Type I-E system.

## Outlook

It is expected that all essential steps of antiviral defense systems are potential targets for dedicated viral inhibitors, as this will provide selective advantage for the virus. To date, viral suppressors have been discovered for only two CRISPR-Cas subtypes, but specific phage-encoded inhibitors most probably exist for all other CRISPR systems as well. This constant huge pressure on CRISPR-Cas systems is an important driving force that would explain their exceptional mutation rates. This rapid evolution is the only way to keep the Red Queen running.

## Abbreviations

Acr: anti-CRISPR
crRNA: CRISPR RNA
R/M: restriction/modification
RNAi: RNA interference
siRNA: small interfering RNA
